# Assessment of faecal sludge quality, heavy metal contamination, and ecological risk: implications for sustainable agriculture

**DOI:** 10.1007/s10661-024-13385-5

**Published:** 2024-11-29

**Authors:** Phillimon T. Odirile, Veronica C. Obuseng, Mohau Moshoeshoe, Lamong Tshenyego, Bontle Mbongwe

**Affiliations:** 1 Department of Civil Engineering, University of Botswana, Private Bag UB0061, Gaborone, Botswana; 2 Chemistry Department, University of Botswana, Private Bag UB0022, Gaborone, Botswana; 3 Department of Chemistry and Chemical Technology, National University of Lesotho, Maseru, Lesotho; 4 Department of Environmental Health, School of Public Health, University of Botswana, Gaborone, Botswana

**Keywords:** Pit latrines, Faecal sludge, Heavy metals, Agriculture, Wastewater treatment, Ecological well-being

## Abstract

Pit latrines represent the predominant form of on-site sanitation in Botswana, posing unique challenges in faecal sludge (FS) management. The key concerns revolve around FS extraction, treatment, and safe disposal. Currently, co-treatment with wastewater is the primary approach, but it strains wastewater treatment plants (WWTPs) and compromises effluent quality. This study comprehensively assesses FS quality from pit latrines and evaluates the potential health risks when used in agriculture for soil improvement/amendment. Systematic sampling of soils at various depth intervals, approximately 30 cm apart, was conducted, followed by extensive laboratory analysis, including determination of heavy metals (copper, iron, lead, cadmium, zinc, manganese, and arsenic) using inductively coupled plasma atomic emission spectroscopy (ICP-OES) and nutrient analysis using ion chromatography (IC). The findings unequivocally demonstrate that FS from VIP (ventilated improved pit) latrines poses no significant health risks due to heavy metal content. Specifically, Geo-accumulation Index (I_geo_) values for nickel (Ni), chromium (Cr), and arsenic (As) were consistently negative, indicating negligible risk of environmental contamination. Copper (Cu) concentrations averaged 40.36 mg/kg in samples collected from Mogoditshane and 591.61 mg/kg in those collected from Broadhurst (Gaborone, Botswana) with Igeo values indicating a moderate pollution risk. Nutrient analysis showed high levels of nitrogen (NO_3_^−^), with concentrations reaching 4.47 × 10^3^ mg/kg in some samples, and phosphorus (PO_4_^3−^) levels as high as 3.9 × 10^4^ mg/kg. These findings highlight its agricultural potential for soil amendment, though prudent management is needed to mitigate eutrophication. The study advocates for separate FS treatment, resolving co-treatment operational challenges and enhancing sustainability. Implementing these recommendations promises to address FS management issues, bolster food security, and enhance Botswana’s ecological well-being.

## Introduction

In pursuit of enhancing agricultural productivity around major settlements in Botswana, the government has allocated funding to support small-scale horticultural projects near the Gaborone Wastewater Treatment Plant (GWWTP) in Gaborone (Odirile et al., 2018). This initiative addresses the challenge posed by the generally poor physical conditions of soils in semi-arid regions like Botswana, characterized by limited water retention capacity and low plant nutrient content (Odirile et al., 2018). The conventional use of commercial fertilizers to bolster agricultural production has proven effective but is accompanied by increased production costs (Hammer & Hammer, 2014). As a sustainable and efficient alternative, the utilization of sludge as fertilizer offers promise in restoring nutrients to agricultural soils (Malkki, 1999).

Wastewater treatment processes generate sludge as a by-product, which consists of organic and inorganic materials separated from the incoming wastewater through a combination of mechanical, biological, and chemical treatments (Hammer & Hammer, 2014). However, this sludge can contain hazardous substances, such as heavy metals, micro-pollutants, and pathogens, posing potential risks to human health and the environment (Gimeno-García et al., 1996; Hashem, 2000).

Studies have demonstrated that using sludge as a soil amendment can elevate concentrations of metals like Cd, Ni, Cu, and Zn in crops such as wheat, potatoes, and leafy vegetables (Jiang et al., 2014). Notably, lead (Pb) tends to remain relatively unavailable to crops from the soil (Wuana & Okieimen, 2011). Additionally, the availability of metals to crops is reportedly lower in soil treated with dried sludge compared to liquid sludge (Zhang et al., 2010).

Recent studies emphasize the importance of assessing heavy metal contamination in faecal sludge due to the potential ecological risks and health impacts. For example, Zewde et al. (2021) highlighted that faecal sludge management practices in low-income areas can introduce high levels of microbial and heavy metal contamination into soils, affecting agricultural sustainability. Similarly, a study by Rahmati et al., (2024a, b) underscored the value of separating faecal sludge treatment from conventional wastewater treatment to improve both effluent quality and sludge reuse potential in agriculture. These findings align with the current research focus on determining the feasibility and safety of using faecal sludge as a sustainable fertilizer in Botswana.

In light of these considerations, human faecal sludge from ventilated improved pit latrines (VIP latrines) emerges as a viable alternative fertilizer due to its rich nutrient content and its ability to enhance soil quality (Nikiema et al., 2013). While human faeces have been recognized as a valuable nutrient source in several countries worldwide, including China, Japan, Korea, and various African and South American nations, its acceptance in Botswana remains limited, primarily utilized by select urban residents for landscaping and gardening purposes (Jönsson et al., 2004). Nevertheless, it is essential to acknowledge that faecal material represents a critical threat to human and animal health, as well as ecosystem integrity (Graham & Polizzoto, 2013).

Pit latrines are a global sanitation solution, serving approximately 1.77 billion people as their primary sanitation method (Diener et al., 2014). They offer cost-effective, water-efficient, and low-maintenance sanitation, particularly valuable in water-scarce regions such as Botswana (Dzwairo et al., 2006). The utilization of pit latrines has significantly improved sanitation conditions in developing countries, particularly in preventing parasitic and bacterial infections among children and infants (Carr & Strauss, 2001).

However, the management of faecal sludge, including pit emptying, transport, treatment, and disposal, poses complex challenges (Graham & Polizzoto, 2013). According to previous research, the annual quantity of sludge generated from pit latrines averages around 520 kg per person, primarily comprising urine and faeces (Jacks et al., 1999). Urine is rich in nitrogen, while faeces contain substantial phosphorous and potassium levels (Jönsson et al., 2004). Recycling this sludge into the soil can replenish these essential nutrients, sustaining land fertility and agricultural productivity (Jacks et al., 1999). Moreover, faecal sludge boasts a low content of heavy metals, a marked contrast to inorganic fertilizers, which frequently contain elevated heavy metal levels (Nziguheba & Smolders, 2008). Notably, phosphate fertilizers, widely used in agriculture, often contain arsenic (As), cadmium (Cd), and lead (Pb) as inherent components of phosphate rock ore or other ingredients, resulting from the phosphate fertilizer industry’s processes (Macedo et al., 2009). The application of faecal sludge to soil, therefore, poses minimal threats related to heavy metal pollution, assuming it remains uncontaminated by industrial wastewater (Nziguheba & Smolders, 2008).

The current sanitation management paradigm involves the emptying of pit latrines, sludge transportation, and subsequent disposal, treatment, or reuse. Notably, heavy metal pollution within faecal sludge poses potential risks to human health, as these pollutants can be transferred to humans through crop consumption. Furthermore, the long-term application of untreated sludge on farmlands can diminish soil buffering capacity, thereby jeopardizing ecological environments (Jiang et al., 2014).

The primary aim of this study is to evaluate the chemical properties of faecal sludge and to assess the potential risks and benefits associated with the reuse of VIP sludge in agriculture. The investigation encompasses the determination of metal content using inductively coupled plasma-optical emission spectrometry (ICP-OES) and the analysis of nutrients, including nitrate (NO_3_^−^), nitrites (NO^2−^), and phosphates (PO_4_^3−^), using ion chromatography (IC) (Jiang et al., 2014). The collected data will offer critical insights into the feasibility and safety of utilizing VIP sludge as a valuable resource within the context of Botswana’s agricultural and environmental sustainability.

### Research context and novelty

The study focuses on Botswana, a region with limited research on faecal sludge quality, heavy metal pollution, and ecological risk. While faecal sludge has been studied in various contexts, its quality, heavy metal content, and ecological implications have not been extensively explored, especially in relation to agricultural use. The detailed assessment of heavy metal concentrations and their potential ecological risks in faecal sludge is a unique aspect of this study, shedding light on a less-explored area of environmental concern. This research explores the potential of using faecal sludge as a sustainable agricultural resource, aligning with the increasing interest in eco-friendly farming practices and resource optimization (Zewde et al., 2021).

## Study area

**Fig. 1:**
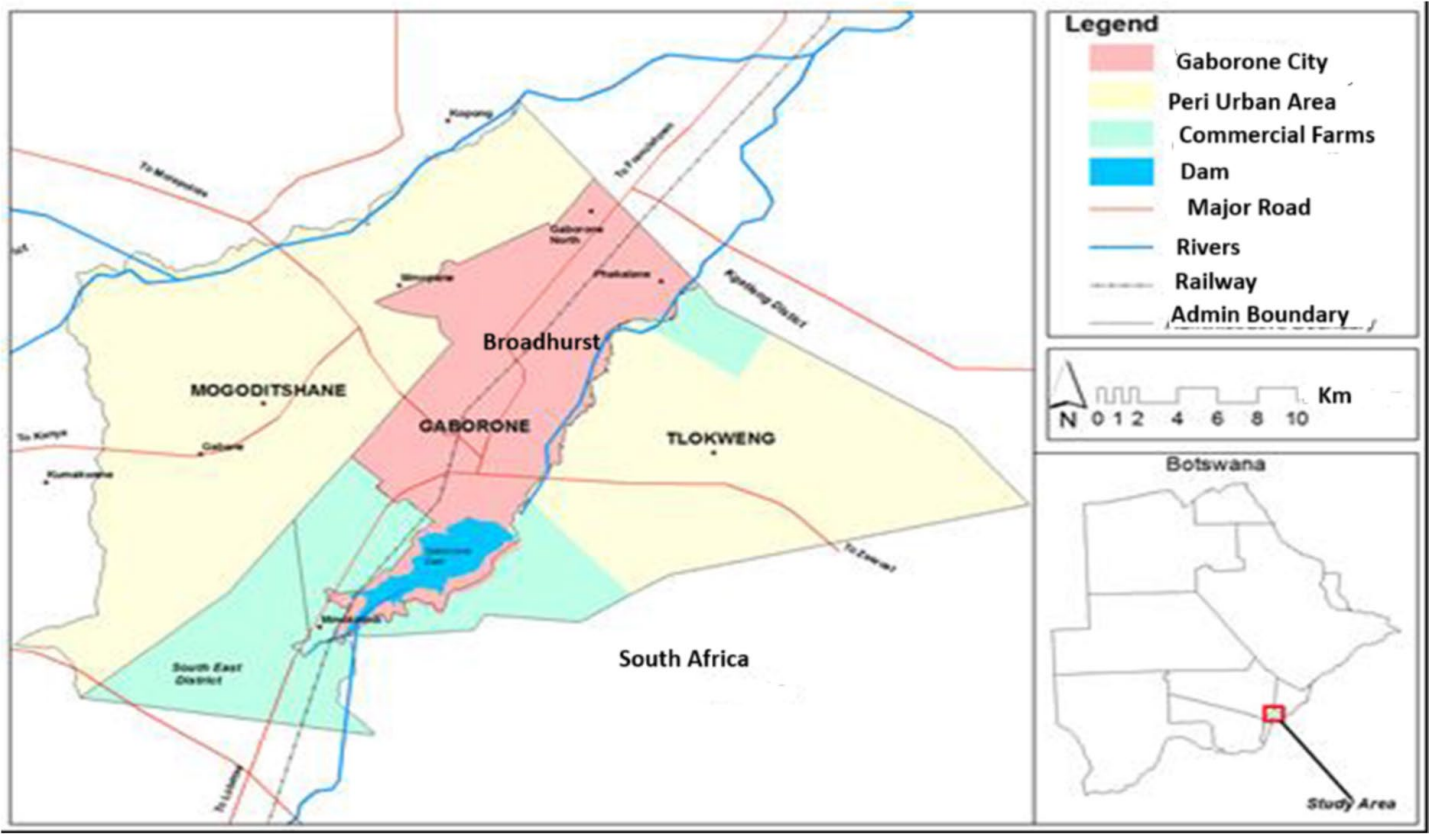
The study area. Insert location of Mogoditshane and Broadhurst relative to Gaborone, Botswana

This study was conducted within the borders of Botswana, a landlocked country situated in southern Africa. Botswana is geographically surrounded by Namibia to the west, Zambia to the north, Zimbabwe to the northeast, and South Africa to the south, as depicted in Fig. 1.

The research specifically focused on two distinct regions within the capital city of Botswana, Gaborone, namely Mogoditshane and Broadhurst. These two areas were selected to represent varying socio-economic settings within the city. Mogoditshane, characterized by an unplanned rural village setup, stands as an exemplar of community-driven pit latrine construction with limited supervision. In Mogoditshane, the pits typically range in depth from 1.3 to 2.8 m, with an average depth of approximately 1.83 m. On the other hand, Broadhurst, located in a peri-urban context, presents a different scenario. The depth of sludge within the pits of Broadhurst depends on the duration of toilet use.

Gaborone serves as both the capital and the most populous city in Botswana, boasting a population of approximately 231,626 residents according to the 2011 census, constituting about 10% of the nation’s total population. The geographic coordinates of Gaborone City are situated at 24° 40′ south latitude and 25° 55′ east longitude. Notably, faecal sludge originating from Mogoditshane and Broadhurst undergoes core treatment at the Gaborone Wastewater Treatment Plant, situated approximately 10 km northeast of Gaborone City, Botswana.

## Materials and methods

### Sludge sample collection and preparation

**Fig. 2:**
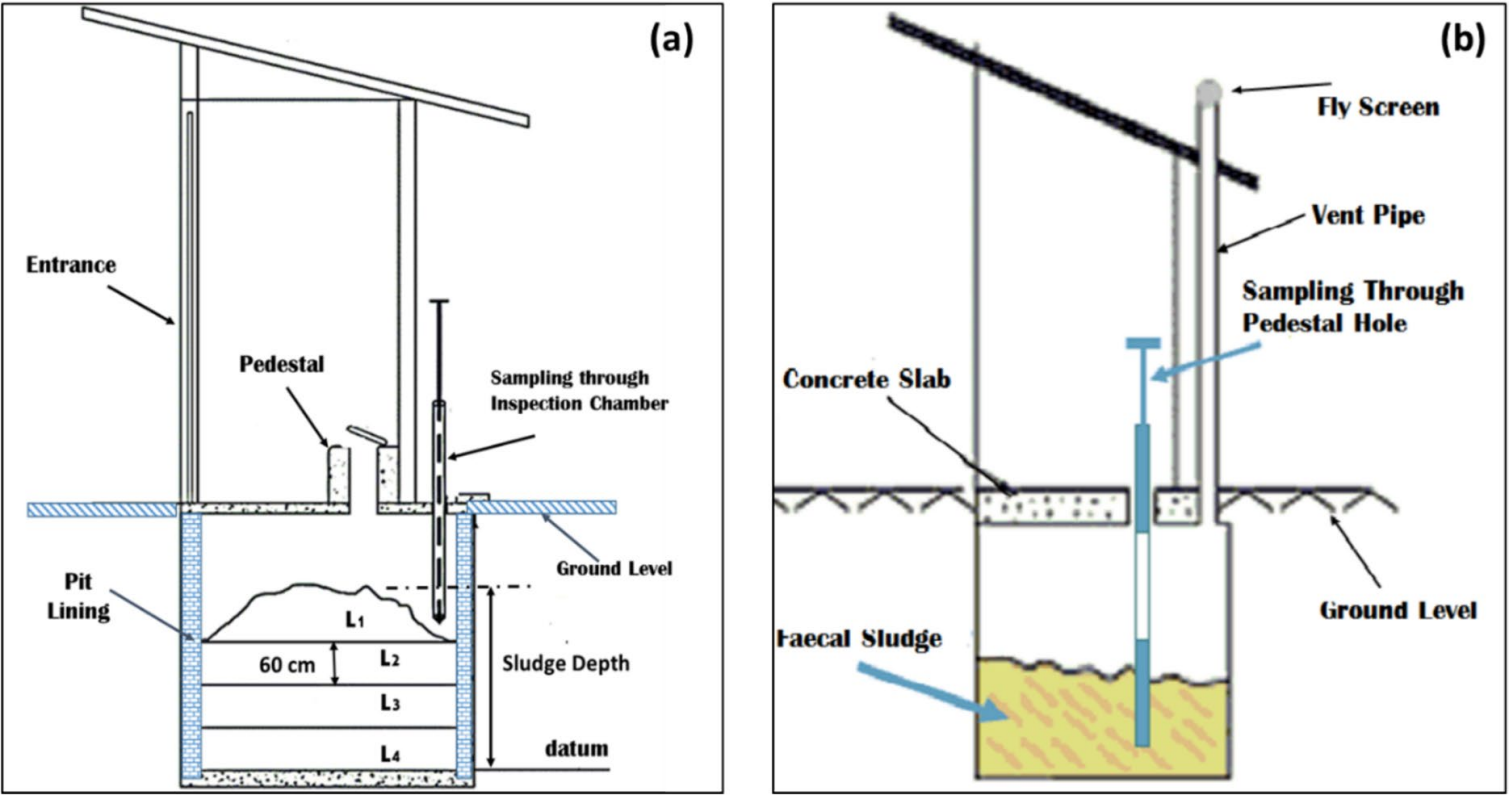
VIP latrine showing FS sampling procedure through the **a** inspection chamber and through the **b** Pedestal. L denotes the FS layer, where L1 is the newest sludge and L4 is the oldest

A total of 50 pits were sampled from two localities in Gaborone: 25 pits in Mogoditshane and 25 pits in Broadhurst, representing different socio-economic contexts. Sludge samples were collected at 60-cm depth intervals, referred to as “Layers.” Due to the shallower sludge accumulation in Mogoditshane pits, only two layers were sampled, whereas the deeper pits in Broadhurst allowed for sampling of four layers. Samples were extracted using a Multistage Sludge Sampler, either through the pedestal hole or the inspection chamber, as shown in Fig. 2.

Sludge samples (about 3 kg) were collected in polyethylene containers and placed in a cooler box immediately after measurement of temperature and pH. They were then transported to the cold room at the University of Botswana in the Chemistry Department, where they were stored at a temperature of 4 °C. For chemical analysis, samples were taken from the cold room and air-dried for 5 days to let water evaporate from the sample and also to avoid microbial action. After drying the samples, they were crushed with pestle and mortar and sieved to a size of 150 µm for particle size homogeneity and easy sample dissolution. The sieved samples were thereafter stored in sealed bottles at room temperature until the day of analysis.

### Analytical methods

#### Metal analysis by ICP-OEP

Faecal sludge samples were securely transported to the Water and Environmental Engineering Laboratory of the University of Botswana and later to the Chemistry where they were analyzed for heavy metals (such as copper (Cu), iron (Fe), lead (Pb), cadmium (Cd), zinc (Zn), manganese (Mn), and arsenic (Ar)) and microbial parameters (such as Ascaris), respectively. All analyses of faecal sludge samples were conducted following the methods outlined by APHA/AWWA/WEFW (2005) and Reddy (2013). Faecal sludge sampling protocols were followed carefully to prevent contamination during sampling and transportation which may affect the results. The protocols used in the study were approved by the Departmental Ethics Committee of the University of Botswana.

All reagents used were of analytical grade, and deionized water (18.2 MΩ cm) was sourced from a Millipore Milli-Q system.

The samples were heated until the solution volume was reduced to approximately 0.5 mL. Samples were then cooled to about room temperature. After digestion, the residue was allowed to air-cool, and then, DDW was added. The obtained solution was filtered through a Whatman No. 42 filter paper and quantitatively transferred to a 25 mL volumetric flask and diluted with DDW to a final volume of 25 mL. Each sample was analyzed in triplicate for Cd, Cr, Cu, Ni, Pb, Mn, Sn, Fe, Zn, As, Na, K, Mg, and Ca using ICP-OES. The results shown here represent layers one and two.

All reagents used were of analytical grade and deionized water (18.2 MΩ cm) from a Millipore Milli Q system. All the extraction procedures were performed using laboratory glassware and polyethylene bottles pre-cleaned with HCl and rinsed with double distilled water.

### Data analysis

The study analyzed the results by calculating the means, standard deviations, and *P-*values for both faecal sludge samples collected from peri-urban and rural areas. Microsoft Office Excel 2007 was used for this data analysis.

To evaluate the extent of heavy metal pollution in faecal sludge, the Geo-accumulation Index (Igeo) proposed by Müller (1979) was employed, as depicted in Eq. 1:

1 $${I}_{geo}=Log\; 2\left[\frac{{C}_{n}}{1.5{B}_{n}}\right]$$

where *C*_*n*_ is the measured heavy metal concentration in mg/kg and *B*_*n*_ is the geochemical background value, mg/kg. To determine the pollution level of the sample, the Geo-accumulation Index is classified in Table 1. A factor of 1.5 is introduced to minimize the potential impact of variations in the background values, which may result from lithologic differences in the sediments (Müller, 1979; Nowrouzi & Pourkhabbaz, 2014).

**Table 1:** Contamination categories based on Geo-accumulation Index (Nowrouzi and Pourkhabbaz (2014))

Index	Category	Description
Geo-accumulation Index (*I*_*geo*_)	≤ 0	Practically unpolluted
	> 0 to ≤ 1	Slightly polluted
	> 1 to ≤ 2	Moderately polluted
	> 2 to ≤ 3	Moderately to strongly polluted
	> 3 to ≤ 4	Strongly polluted
	> 4 to ≤ 5	Strongly to very strong
	> 6	Very strong

### Compliance with ethical standards

This study was conducted in compliance with ethical guidelines provided by the University of Botswana Office of Research and Development (ORD). All procedures involving sample collection, handling, and analysis were reviewed and approved by the ORD to ensure adherence to ethical standards and to safeguard the health and safety of researchers and the community. In addition, informed consent was obtained from all participants involved in the study, and efforts were made to ensure the confidentiality and privacy of data.

## Results and discussions

### Nutrient content in FS

The effects of organic matter, nitrogen, phosphorus and toxic elements in sewage sludge applied to agricultural land have been reviewed extensively in the literature. However, that effect is still limited in terms of pit latrine/faecal sludge which is rich in organic matter and may improve the structure and water holding capacity of poor soils as well as containing agronomically significant amounts of nitrogen and phosphorus in sludge to render it of fertilizer value (Malkki, 1999).

**Fig. 3:**
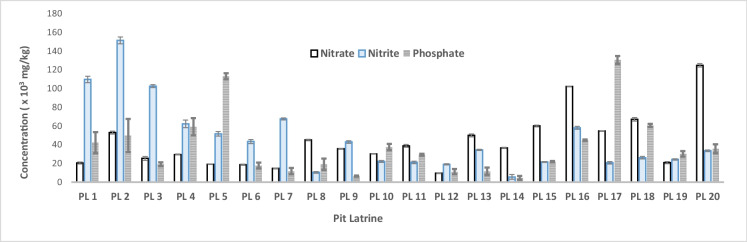
Mean amounts of nitrate, nitrite, and phosphate in various pit latrines as sampled in Mogoditshane village in Botswana. PL 1 to PL 20 denote pit numbering/labels. Means were calculated from replicates of extraction and analysis

Results in Fig. 3 and Table 2 revealed that the amounts of NO_3_^−^, NO_2_^−^, and PO_4_^3−^ in the pit latrine sludges studied were very high. For example, in Mogoditshane, nitrate concentrations reached as high as 4.47 × 10^4^ mg/kg. These are important nutrients in crop production. The results show that in all pits investigated in Mogoditshane, average nitrite concentrations were higher than nitrate concentrations, but this was reversed in samples collected from Broadhurst pits. The average phosphate concentration in the two sampling areas was comparable, as high as 39,000 mg/kg. Following the principles of sustainable development, nutrients in faecal sludge should be used in plant production, instead of ending up in wastewater treatment plants. However, the most undesirable consequence of such high nutrient concentrations is the risk of pollution which is posed by pit latrines. Several researchers have found that pit latrines are a source of nitrate contamination and therefore a hazard to groundwater due to their huge capacity to play a part in chemical and/or microbial pollution (Appiah-Effah et al., 2015; Gimeno-García et al., 1996; Graham & Polizzoto, 2013; Jacks et al., 1999; Jönsson et al., 2004; Mafa, 2003; Nikiema et al., 2013). Earlier studies carried out by Mafa (2003) in the city of Francistown in Botswana showed that pit latrines were found to have the highest impact on groundwater quality, resulting in such groundwater being unsuitable for consumption. Nitrogen (in the form of nitrate) is the most dominant of all these and is therefore used as a key indicator of overall groundwater quality (Graham & Polizzoto, 2013; Jacks et al., 1999; Jönsson et al., 2004). Moreover, it has been shown that in Botswana about 50% of nitrogen from pit latrines leaches to groundwater (Jacks et al., 1999).

**Table 2:** Minimum, maximum, and average values of NO_3_^−^, NO_2_^−^, and PO_4_^3−^ in pit latrine sludge sampled from Mogoditshane and Broadhurst

Analyte (× 10^3^ mg/kgdwt)	Location	Minimum (mg/kgdwt)	Maximum (mg/kgdwt)	Mean ± SD (mg/kgdwt)
(NO_3_^−^)	Mogoditshane	9.93 ± 0.14	125.22 ± 1.14	42.47 ± 3.21
	Broadhurst	6.06 ± 0.19	92.87 ± 3.03	46.33 ± 1.31
(NO_2_^−^)	Mogoditshane	5.96 ± 0.21	159.01 ± 3.75	59.61 ± 2.62
	Broadhurst	9.77 ± 3.19	149.03 ± 7.16	33.73 ± 2.03
(PO_4_^3−^)	Mogoditshane	4.28 ± 0.79	134.22 ± 2.28	38.02 ± 1.13
	Broadhurst	5.56 ± 0.28	121.09 ± 6.59	39.52 ± 6.22

The results from all the pits were pooled and averaged per sampling location. These results indicated high average concentrations of all the nutrients above recommended maximum level that could contaminate groundwater. In the area where sampling was carried out, there are boreholes in close proximity to several pit latrines. Previous research has shown that such close distances between groundwater sources and pit latrines always lead to chemical and/or microbiological contamination of groundwater (Graham & Polizzoto, 2013; Nikiema et al., 2013), leading to nutrient values above the maximum allowed limit (MAL) of 50 mg/L set by WHO (2011). In addition to the fact that the soils in Botswana are permeable and thus allow for excessive bacterial and chemical pollution (Vogel, 2002), almost all the pits in the area on which we sampled from were not lined internally, thereby presenting little or no impediment to the mobility of bacteria and other faecal contaminants. The observed high variability (between minimum and maximum amount) could be attributed to poor mixing within layers as the faecal sludge was observed to be thick.

The results demonstrate that FS from VIP latrines contains high levels of nitrogen and phosphorus, essential for soil fertility in semi-arid regions. These findings are consistent with earlier research by Nikiema et al. (2013), who emphasized the nutrient-rich potential of faecal sludge for agricultural use. Another notable finding is the high concentration of nitrogen (NO_3_^−^) in FS, with values reaching 4.47 × 10^3^ mg/kg in Mogoditshane. While this suggests strong fertilizing potential, it also poses a risk of groundwater contamination, particularly in areas where pit latrines are located near water sources. These results are consistent with studies by Jacks et al. (1999) and Graham and Polizzoto (2013), which documented the risks of nitrogen leaching from pit latrines, further emphasizing the need for careful management practices to prevent water pollution.

#### Risk of heavy metal in agricultural soils

In most cases, heavy metal contamination of soils is due to the release into the environment from activities such as mining, animal manure, petrochemical spillages, paints, and wastewater treatment sludge application to soils (Wuana & Okieimen, 2011). Heavy metals commonly found at contaminated sites include the following: lead (Pb), chromium (Cr), copper (Cu), zinc (Zn), cadmium (Cd), arsenic (As), nickel (Ni), and mercury (Hg) (Wuana & Okieimen, 2011) to name just a few. These metals do not undergo microbial or chemical degradation as it is the case with organic contaminants which are oxidized to carbon (IV) oxide by microbial action. The heavy metals also inhibit the biodegradation of organic compounds (Wuana & Okieimen, 2011).

**Fig. 4:**
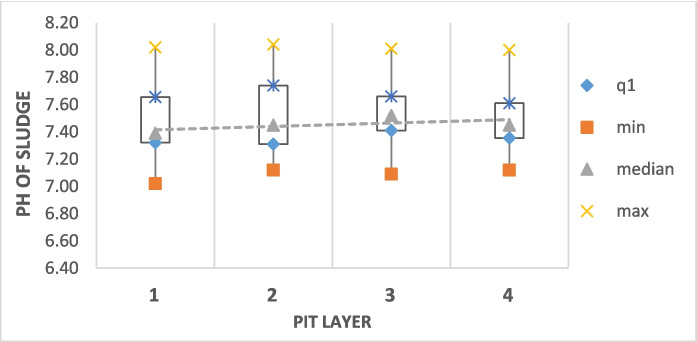
Average pH values for pit sludge for all 50 pits from all the Gaborone areas of Mogoditshane and Broadhurst. The pH was taken on-site

The most hazardous to humans among heavy metals in sludge are cadmium, mercury, and lead, while copper, zinc, chromium, and nickel. In high concentrations, these metals are particularly poisonous to plants (Hashem, 2000). The data from the two areas of Mogoditshane (Tables 3 and 4) show average heavy metal content in faecal sludge is generally low as a potential risk to humans. The results, however, show escalated amounts of copper compared to the South African limits for spreading of sludge on fields. However, pH has an effect on the behaviours of these metals. Metals are bound to soils at a pH exceeding 6.5 and/or with a high organic matter content. If the pH is below this value, if organic matter is consumed or if all feasible soil adsorption sites are saturated, metals become mobile and can be absorbed by crops and contaminate water bodies (Wuana & Okieimen, 2011). The FS sample results shown in Fig. 4 show that the sludge pH ranges between 7 and 8. This suggests that the pH may enhance metal binding in soils once the sludge is applied.

**Table 3:** Heavy metal concentrations in faecal sludge from 25 Mogoditshane pits

Metals	Mogoditshane metals (mg/kg)					
	Layer 1		Layer 2		Limits for spreading on fields	
	Mean	SD	Mean	SD	South Africa	European Union
Ni	8.62	0.33	9.33	0.31	200	300–400
Cr	28.72	2.07	39.63	1.91	1750	1000–1500
Pb	9.31	0.42	8.29	0.35	50.5	750–1200
Zn	390.59*	5.23	327.55	6.19	353.5	2500–4000
As	1.68	0.14	3.82	0.31	15	-
Cd	0.32	0.02	0.39	0.06	15.7	20–40
Cu	40.36	0.35	43.03	0.38	50.5	1000–1750
Mn	199.83	7.01	204.07	8.77	-	-
Fe	8483	421.17	11854.66	557.76	-	-
Ca	15278.11	2.15	3254.28	2.13	-	-
Sn	1.79	0.14	2.15	0.46	-	-
Na	2114.77	53.97	2254.72	114.95	-	-
Mg	3406.3	163.29	2831.97	137.44	-	-
K	3950.4	52.79	3863.72	74.5	-	-

*Exceed the South African limits for spreading on fields. -, no limits; SD, standard deviation

**Table 4:** Faecal sludge heavy metal concentrations for 25 Broadhurst pits relative to the South Africa and European Union limits for spreading on agriculture

Metals	Broadhurst metals (mg/kg)									
	Layer 1		Layer 2		Layer 3		Layer 4		Limits for Spreading on field	
	Mean	SD	Mean	SD	Mean	SD	Mean	SD	South Africa	European
										Union
Ni	3.34	0.08	2.02	0.05	1.88	0.04	2.53	0.78	200	300–400
Cr	13.43	0.6	10.78	0.42	15.15	0.69	8.65	3.94	1750	1000–1500
As	0.83	0.14	6.32	0.3	0.87	0.19	7.28	3.28	15	-
Cd	0.19	0.01	0.3	0.02	1.26	0.02	0.96	0.96	15.7	20–40
Cu*	591.61*	37.72	672.15*	49.76	733.17*	30.67	455.08*	26.21	50.5	1000–1750
Sn	0.64	0.11	1.47	0.43	1.03	0.24	11.69	4.76	-	-

*Exceed the South African limits for spreading on fields.-, no limits; SD, standard deviation

An important consideration in the application of sludge to farmland is its limitation of possible addition of toxic elements and any beneficial effects are secondary to this. This is because crops can accumulate toxic elements from sludge-amended soils and where heavily contaminated sludges and excessive rates of application are used plants may accumulate concentrations which are toxic to plants. Among the heavy metals in the analyzed sludge, the most hazardous ones to humans are cadmium and lead. However, copper, zinc, chromium, and nickel in high concentrations are particularly poisonous to plants (Reid, 2013).

Heavy metal concentrations in FS are at acceptable levels compared to South African (SA) and European Union (EU) standards for spreading in the fields, except for Cu which exceeds SA limits (Commission Regulation (EU) 2023/915, 2023). It is observed though that the SA limits for Cu are a lot more stringent compared to the EU limit for the same (Dikinya et al., 2011). The Cu levels in Table 3 by far exceed SA limits in all pit levels. However, these values are below EU limits. This indicates that, although both higher in Broadhurst results than the results for both Mogoditshane and Broadhurst areas, the risk posed by the presence of heavy metals in soils is generally low in FS as compared to what is normally found in sewage treatment sludge (Nowrouzi, & Pourkhabbaz, 2014). This suggests that the land application of treated FS is much more viable than the land application of treated sewage sludge. However, environmental concerns regarding land disposal such as surface-water and groundwater pollution and transmission of human and animal diseases should never be taken lightly.

While soil characterization would provide an insight into heavy metal speciation and bioavailability, an attempt at remediation of heavy metal contaminated soils would entail knowledge of the source of contamination, basic chemistry, and environmental and associated health effects (risks) of these heavy metals. In Tables 3 and 4, a range of heavy metal concentrations were found as 0.03–20.447 mg/kg and 0.04–1.513 mg/kg for Mogoditshane and Broadhurst, respectively. For all the two study areas, the concentrations are in the order of Cd ˃˃ As ˃˃Sn ˃˃ Ni Pb ˃˃ Cr ˃˃ Cu ˃˃ Mn ˃˃ Zn ˃˃ Fe. Although heavy metals are present in pit sludges tested, their concentrations are not posing any risk especially to plants. Therefore, the presence of heavy metals in FS is not significant when compared to their presence in sewerage treatment sludge (STS) (Tytła, 2019). In that case, the application of treated FS to agricultural land should be encouraged rather than the application of STS to agricultural soils.

Sewage sludge is known for its composition rich in organic matter (OM) and essential biogenic compounds, particularly nitrogen (N) and phosphorus (P), crucial for promoting plant growth (Hammer & Hammer, 2014; Hashem, 2000; Wang et al., 1999). Nevertheless, it also contains heavy metals, including toxic ones such as cadmium (Cd), chromium (Cr), copper (Cu), mercury (Hg), nickel (Ni), lead (Pb), and zinc (Zn) (Gimeno-García et al., 1996; Malkki, 1999). The presence of these heavy metals in sewage sludge implies that, depending on their concentration and duration of exposure, they can potentially pose environmental and health hazards, primarily because of their capacity to bioaccumulate within the food chain (Zhang et al., 2010).

The primary sources of heavy metals in sewage sludge encompass domestic and industrial wastewater discharges, sewerage system corrosion, and runoff from urbanized regions or roadways (Wuana & Okieimen, 2011). In light of the elevated heavy metal concentrations found in sewage treatment sludge, the application of treated faecal sludge (FS) for enhancing food production warrants serious consideration. This approach not only mitigates concerns related to the co-treatment of FS with wastewater, which can lead to operational issues at wastewater treatment plants (WTPs), but also presents a more sustainable and business-savvy solution for FS management, ultimately contributing to improved food security.

The significant nutrient content observed in FS samples from our study area positions it as an excellent candidate for land application in agriculture. However, it is important to be mindful of potential runoff issues in areas near surface water bodies where FS has been applied, as the high nutrient content could contribute to eutrophication problems. To mitigate these impacts, the application of sludge should be coupled with best ploughing practices (BPP) to enhance soil retention and prevent nutrient runoff. It is reassuring to note that, in accordance with international standards, the concentrations of heavy metals in FS remain within acceptable limits, further endorsing the feasibility of using FS in agriculture.

The study revealed that faecal sludge (FS) from VIP latrines in Botswana contains high levels of essential nutrients like nitrogen and phosphorus, important for soil fertility in semi-arid regions, aligning with prior research (Nikiema et al., 2013). The low concentrations of heavy metals such as nickel, chromium, lead, and arsenic further support FS’s safe use in agriculture, consistent with findings by Zewde et al. (2021). However, moderate levels of copper raise concerns about long-term accumulation in soils. While copper levels are below European Union limits, they exceed South African standards, posing potential risks if FS is applied continuously, as highlighted by Rahmati et al., (2024a, b) and Nziguheba and Smolders (2008). Careful monitoring is advised to prevent soil contamination.

### Geo-accumulation Index calculation

**Fig. 5:**
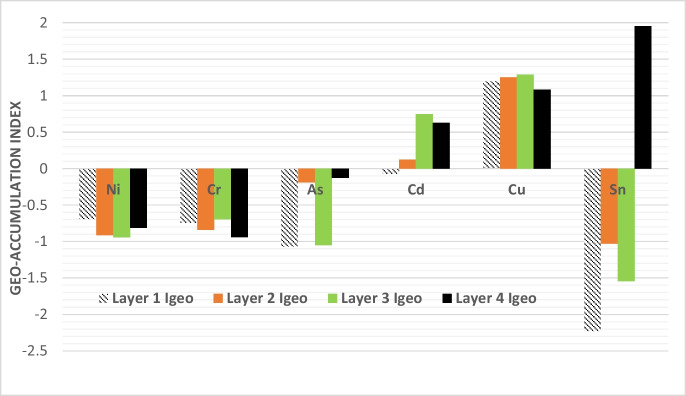
The Geo-accumulation Index of heavy metals in pit latrine sludge at various sludge levels

The Geo-accumulation Index (Igeo) was calculated for the heavy metals analyzed in this study, and the results are presented in Table 5 and Fig 5. The Igeo values provide insights into the potential ecological risk associated with these heavy metals. For nickel (Ni), chromium (Cr), and arsenic (As), the Igeo Index values were found to be below zero, indicating a negligible risk of environmental contamination by these elements. Specifically, Ni, Cr, and As exhibited negative Igeo values across all layers (L1, L2, L3, and L4), emphasizing their minimal impact on environmental pollution.

**Table 5:** Geo-accumulation Index for heavy metals

Metal	*B*_*n*_	Layer 1		Layer 2		Layer 3		Layer 4	
		*C*_*n*_	*I*_*geo*_	*C*_*n*_	*I*_*geo*_	*C*_*n*_	*I*_*geo*_	*C*_*n*_	*I*_*geo*_
Ni	22	3.34	− 0.694	2.02	− 0.912	1.88	− 0.943	2.53	− 0.814
Cr	100	13.43	− 0.747	10.78	− 0.842	15.15	− 0.695	8.65	− 0.938
As	13	0.83	− 1.070	6.32	− 0.188	0.87	− 1.049	7.28	− 0.127
Cd	0.3	0.19	− 0.073	0.3	0.125	1.26	0.748	0.96	0.630
Cu*	50	591.61	1.198	672.15	1.253	733.17	1.291	455.08	1.084
Sn	2	0.64	− 2.228	1.47	− 1.029	1.03	− 1.545	11.69	1.958

In the case of chromium (Cr), there was a slight indication of potential pollution within layers L3 and L4, as suggested by the Igeo values slightly below zero. However, this risk was still considered low and may not significantly affect the environment. Conversely, copper (Cu) exhibited Igeo values above zero, indicating a moderate risk of pollution. These values were consistently below 2, signifying that while Cu poses some risk, it remains within manageable limits. Figure 5 illustrates the Geo-accumulation Index values of these heavy metals across different sludge layers, providing a visual representation of their ecological implications.

Overall, the Geo-accumulation Index (Igeo) calculations emphasize that the heavy metal content in pit latrine sludge, especially for Ni, Cr, and As, does not pose substantial environmental risks. However, continued monitoring and proper management practices are advisable to mitigate potential pollution concerns, particularly in the case of copper (Cu). Therefore, Igeo values suggest the need for careful management to avoid excessive accumulation in soils, which could impair long-term agricultural sustainability. This aligns with Rahmati et al., (2024a, b), who noted that heavy metal buildup in agricultural soils can pose significant environmental risks. Similarly, Nziguheba and Smolders (2008) also found that copper, in particular, poses potential risks when sludge is used as a soil amendment.

The statistical analysis of sludge characteristics between Mogoditshane and Broadhurst revealed significant differences across all measured parameters. *P*-values well below the significance threshold of 0.05, ranging from 4.20 × 10^−19^ to 4.07 × 10^−101^, confirm substantial disparities in nutrient and heavy metal concentrations between the two regions. The large *T*-statistics further emphasize the magnitude of these differences. Notably, higher levels of nitrites, copper, and zinc in Broadhurst compared to Mogoditshane indicate distinct environmental conditions and sludge composition. These findings underscore the importance of developing region-specific strategies in environmental management to effectively address the unique risks and opportunities associated with using faecal sludge in agriculture and waste management.

## Implications and future research directions

The findings of this study have several important implications for sustainable agriculture and environmental management. The use of faecal sludge (FS) as a soil amendment shows great potential due to its high nutrient content, particularly nitrogen and phosphorus. This could play a critical role in improving soil fertility in semi-arid regions like Botswana, where soil nutrient depletion is a major challenge for agricultural productivity. However, the moderate levels of copper (Cu) observed in FS highlight the need for careful monitoring to avoid the risk of heavy metal accumulation in soils, which could have adverse effects on crop health and long-term soil viability. Additionally, the high concentrations of nitrogen (NO_3_^−^) present a risk of eutrophication in nearby water bodies if FS is not managed properly during land application.

Future research should focus on long-term field trials to assess the impact of FS application on crop yield, soil health, and potential heavy metal buildup in different soil types. Moreover, studies should explore the effectiveness of different FS treatment technologies, such as biochar or composting, to reduce heavy metal concentrations and improve FS safety for agricultural use. Research into developing guidelines for the safe and optimal use of FS in agriculture, particularly in regions with similar semi-arid climates, would be invaluable. Lastly, more investigation is needed into the social acceptance and practical implementation of FS as a fertilizer in Botswana, to ensure that it becomes a widely adopted practice in sustainable agriculture**.**

## Conclusion and recommendations

This paper offers a fresh perspective on faecal sludge management, emphasizing its potential benefits and environmental concerns in a specific geographical and agricultural context. The findings, therefore, cater to the local context of Botswana, where water scarcity and agriculture are critical issues, making the research relevant and novel in addressing these challenges. The study concludes that faecal sludge is a valuable resource rich in organic matter and essential nutrients for plant growth. However, the high concentrations of nitrate and phosphate in pit latrine sludge pose a significant risk of groundwater pollution. The presence of heavy metals, while generally low, requires careful management to avoid environmental contamination. Based on the findings, the following conclusions and recommendations are provided:

Faecal sludge contains high levels of organic matter, nitrate, and phosphate, making it a potential fertilizer for plant production. However, elevated concentrations of nitrate and phosphate in pit latrine sludge pose a serious risk of groundwater pollution, emphasizing the need for improved management practices. The concentration of heavy metals in faecal sludge samples is generally low, with copper levels exceeding South African limits but remaining below European Union limits and the Geo-accumulation Index calculations indicate minimal environmental risk for nickel, chromium, and arsenic, but a moderate risk for copper suggesting a need for careful monitoring and management. Based on the findings of this research, the following recommendations are suggested:

- Provide enhanced support for faecal sludge management practices in peri-urban and rural areas to minimize environmental and health risks.
- Develop and implement guidelines and best practices for the safe and controlled use of faecal sludge as a fertilizer, taking into account its nutrient content.
- Continue monitoring heavy metal concentrations in faecal sludge and implement measures to mitigate potential environmental pollution.
- Launch awareness campaigns to educate communities about the risks associated with improper faecal sludge disposal and the benefits of adopting sustainable practices.

## Data Availability

No datasets were generated or analysed during the current study.
